# Supplementary material for: Long-read genome assemblies for the study of chromosome expansion: Drosophila kikkawai, Drosophila takahashii, Drosophila bipectinata, and Drosophila ananassae
Source: G3 (Bethesda). 2023 Aug 23;13(10):jkad191. doi: 10.1093/g3journal/jkad191 (PMC10542312; doi:10.1093/g3journal/jkad191)
Supplement: jkad191_Supplementary_Data [file jkad191_supplementary_data.zip › File_S1_G3-2023-404296.docx]

# Supplementary Tables

**Table S1.** Version information and references for the bioinformatics tools used in this study.

**Table S2.** Number of putative Muller Element F scaffolds and the estimated size of the F Element in each *Drosophila* species with RefSeq assemblies.

**Table S3.** Pacific Biosciences read statistics for *D. bipectinata*, *D. kikkawai*, and *D. takahashii* reported by SequelTools.

**Table S4.** Pacific Biosciences read statistics for *D. bipectinata*, *D. kikkawai*, and *D. takahashii* reported by SEQUELstats.

**Table S5.** Total length of the Muller Elements, their estimated gap sizes, and the number of unresolved regions in *D. melanogaster* (A), and in the four Hi-C scaffolded assemblies: *D. ananassae* (B), *D. bipectinata* (C), *D. kikkawai* (D), and *D. takahashii* (E).

**Table S6.** Comparisons of the assembly statistics for the current RefSeq genome assemblies against the Hi-C scaffolded genome assemblies for *D. bipectinata*, *D. kikkawai*, and *D. takahashii*.

# Supplementary Figures

**Figure S1.** Complete and duplicated BUSCO matches in the *D. bipectinata* Hi-C assembly can partly be attributed to the histone gene cluster on scaffold_165 and scaffold_175.

**Figure S2.** Scaffold statistics, BUSCO scores, and snail plots for the *D. ananassae* (A), *D. bipectinata* (B), *D. kikkawai* (C), and *D. takahashii* (D) Hi-C scaffolded genome assemblies.

**Figure S3.** Repeat density of each chromosome arm for the *D. ananassae*, *D. bipectinata*, *D. kikkawai*, and *D. takahashii* Hi-C scaffolded genome assemblies.

# Supplementary Files

**File S1.** Brief description of the supplementary tables, figures, and files for this manuscript.

**File S2.** List of accession numbers for the scaffolds that have been assigned to the Muller Element F in each *Drosophila* RefSeq genome assembly. This Excel file includes 40 worksheets. The data in the "**F_statistics**" worksheet was used to construct Figure 1. The other worksheets in the Excel workbook provide the underlying data for columns E and F of the "F_statistics" worksheet. The "**RefSeq_F_scaffolds**" worksheet includes the accession numbers for the scaffolds that contain F Element genes in each *Drosophila* RefSeq assembly. The "**Dmel_F_genes_scaffolds**" worksheet includes the list of *D. melanogaster* F Element genes and their placements in each *Drosophila* RefSeq assembly. The 36 worksheets labeled with the names of the UCSC Databases (i.e., “**DalbRefSeq1**” ... “**DyakRefSeq3**”) contain the estimated location of each *D. melanogaster* F Element gene in the corresponding *Drosophila* RefSeq assembly. The "**RefSeq_asm_info**" worksheet includes the accession numbers and links to NCBI for each RefSeq assembly.
