# Supplementary material for: Long-read genome assemblies for the study of chromosome expansion: Drosophila kikkawai, Drosophila takahashii, Drosophila bipectinata, and Drosophila ananassae
Source: G3 (Bethesda). 2023 Aug 23;13(10):jkad191. doi: 10.1093/g3journal/jkad191 (PMC10542312; doi:10.1093/g3journal/jkad191)
Supplement: jkad191_Supplementary_Data [file jkad191_supplementary_data.zip › Supplemental_Figures_G3-2023-404296.pdf]

Supplemental Figures

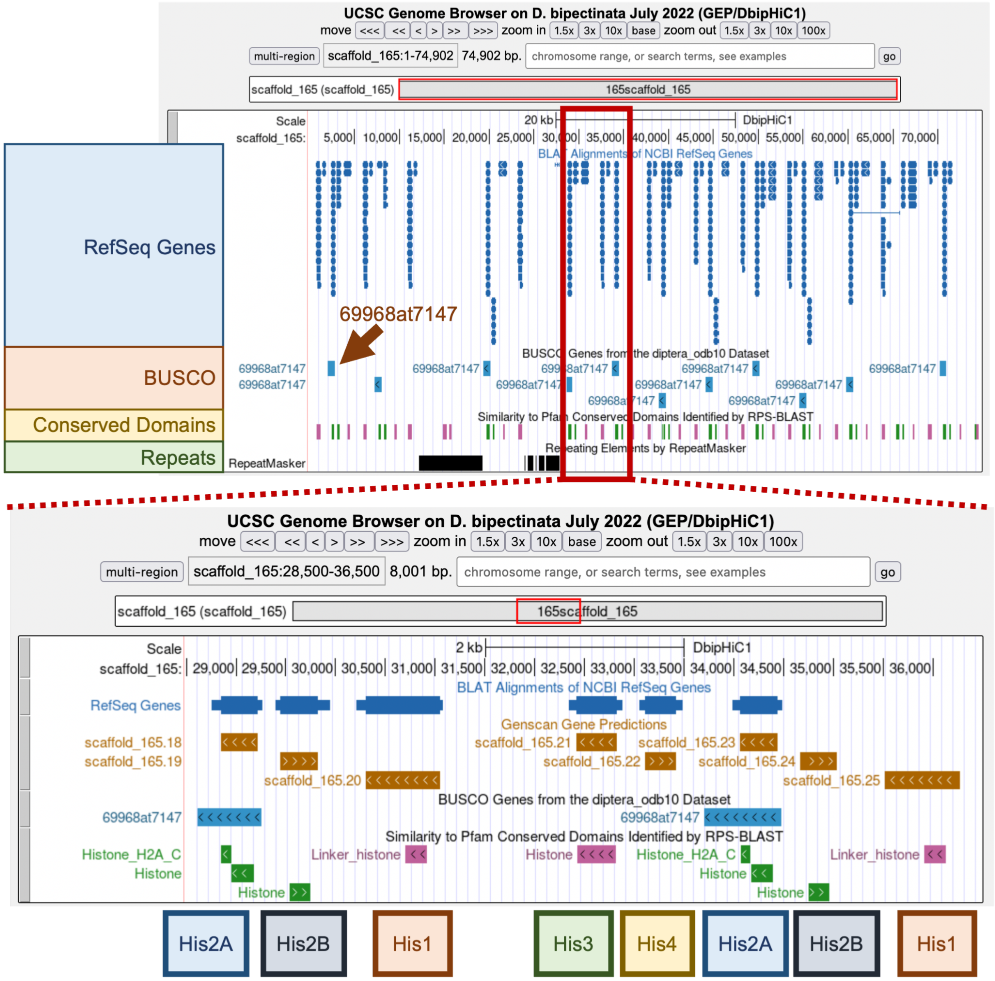

Figure S1 Complete and duplicated BUSCO matches in the *D. bipectinata* Hi-C assembly can partly be attributed to the histone gene cluster on scaffold_165 and scaffold_175. Among the 108 complete and duplicated BUSCO matches in the *D. bipectinata* Hi-C assembly, 29 of them (27%) are located on scaffold_165 and scaffold_175. (Top) Examination of the entire 75 Kb scaffold_165 shows multiple matches to Histone 2A (69968at7147 in the diptera_odb10 lineage dataset; brown arrow). (Bottom) Examination of the 28,500-36,500 region of scaffold_165 shows the histone gene cluster with copies of the genes for Histones 3 (*His3*), 4 (*His4*), 2A (*His2A*), 2B (*His2B*), and 1 (*His1*).

A *D. ananassae* (DanaHiC1)

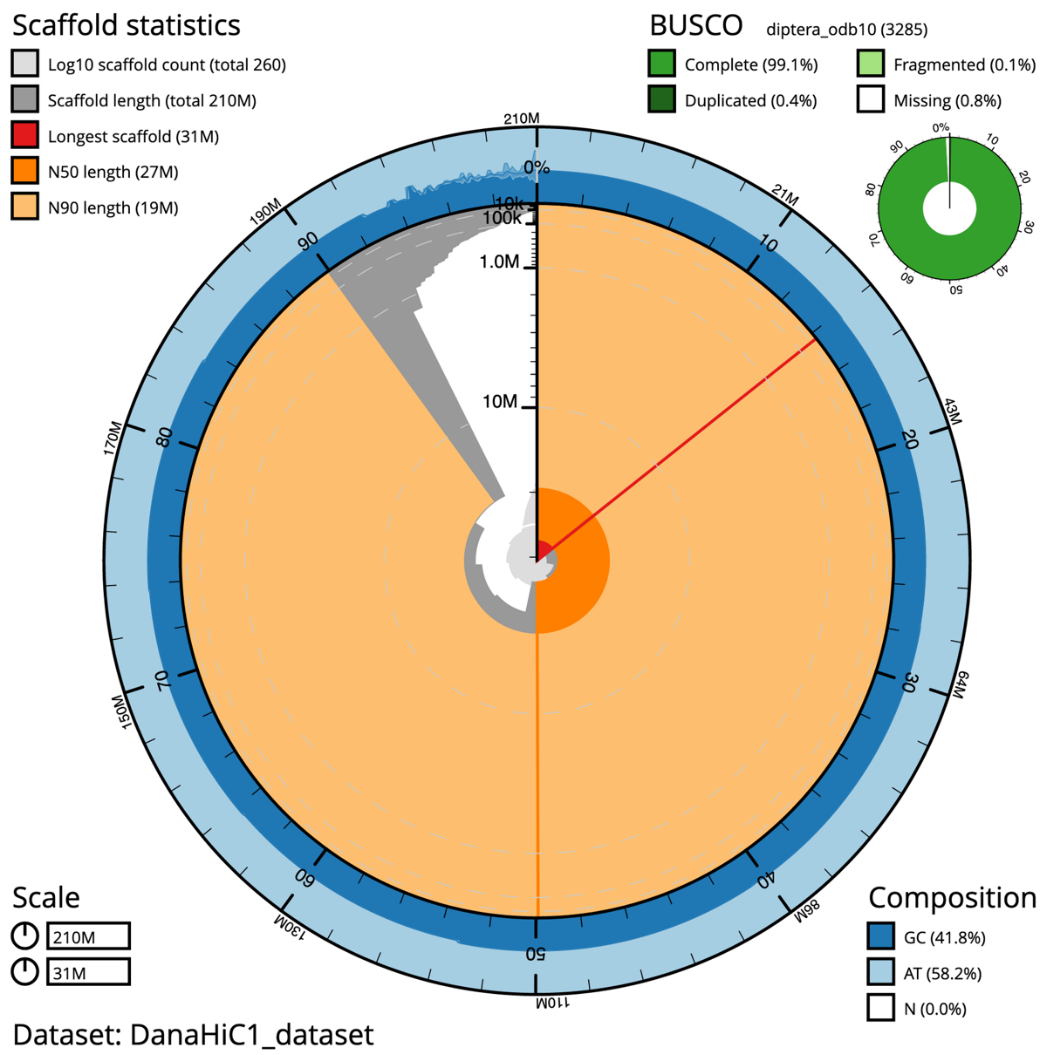

B *D. bipectinata* (DbipHiC1)

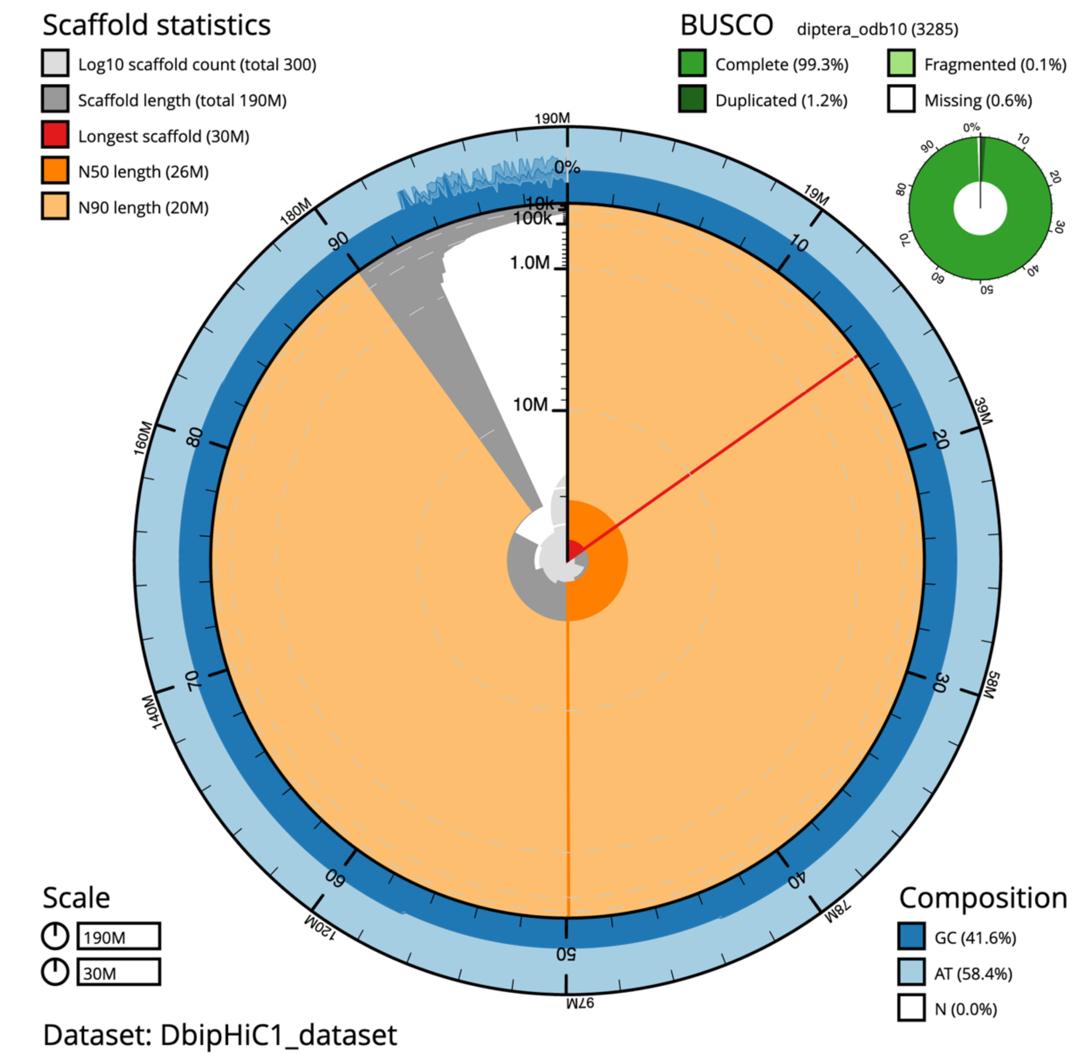

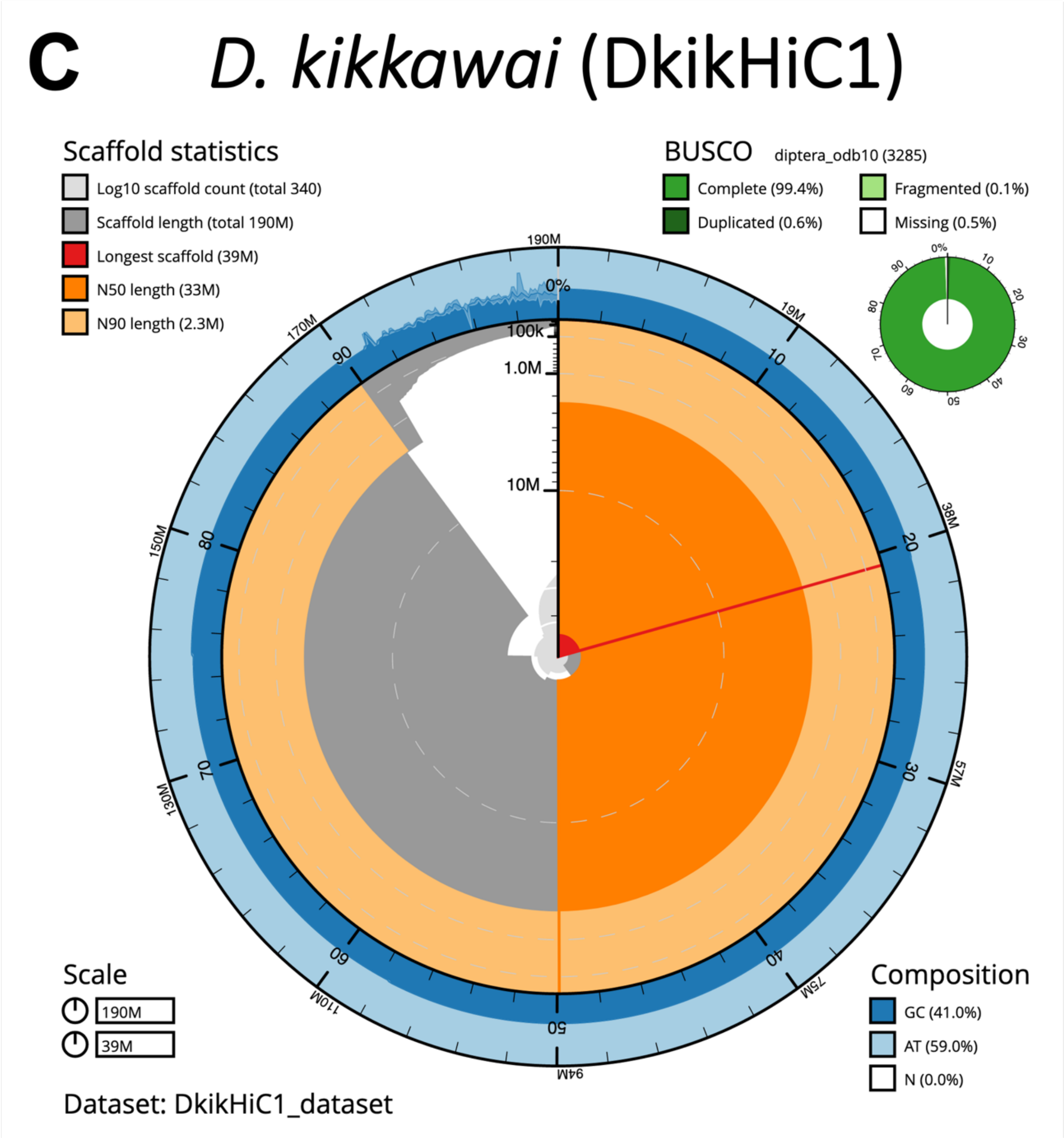
C
D. kikkawai (DkikHiC1)
Scaffold statistics
Log10 scaffold count (total 340)
Scaffold length (total 190M)
Longest scaffold (39M)
N50 length (33M)
N90 length (2.3M)
BUSCO diptera_odb10 (3285)
Complete (99.4%)
Fragmented (0.1%)
Duplicated (0.6%)
Missing (0.5%)
Scale
190M
39M
Composition
GC (41.0%)
AT (59.0%)
N (0.0%)
Dataset: DkikHiC1_dataset

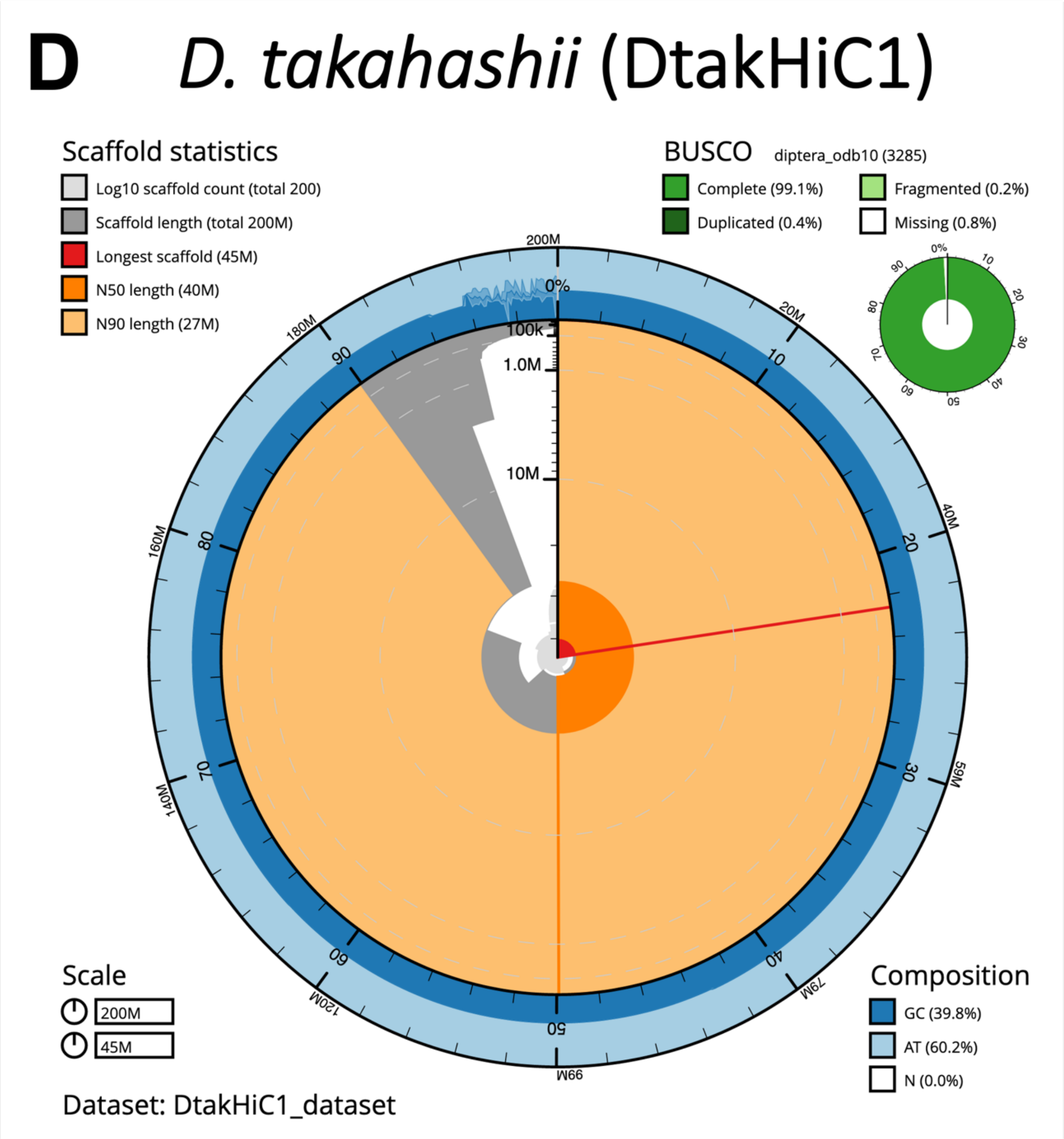

Figure S2 Summary of Hi-C scaffolded assemblies
Snail plots which show the assembly statistics for *D. ananassae* (A), *D. bipectinata* (B), *D. kikkawai* (C), and *D. takahashii* (D). The Hi-C scaffolded assemblies for the four genomes are high quality — with scaffold N50s that range from 26 Mb in *D. bipectinata* to 40 Mb in *D. takahashii* and the percentages of “Complete” BUSCOs range from 99.1% in *D. takahashii* and *D. ananassae* to 99.4% in *D. kikkawai*.

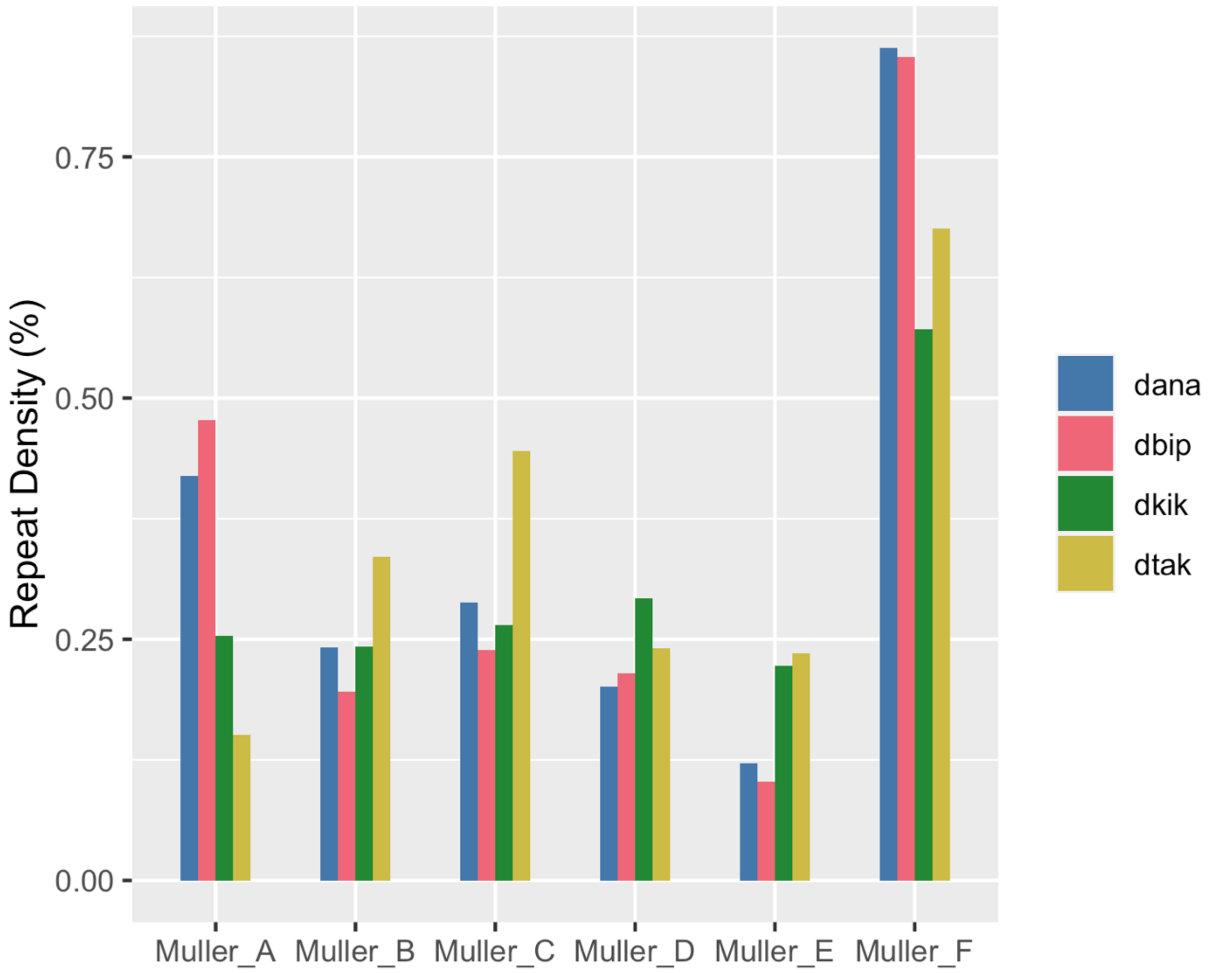

Figure S3 Repeat density per chromosome arm
The percent of total sequences masked by RepeatMasker is shown for each Muller Element, for each species. Muller A and Muller F are larger in *D. ananassae* and *D. bipectinata* (Figure 5) and show higher repeat density, suggesting that their size expansion is driven, at least in part, by accumulation of repetitive elements. Similarly, Muller C is larger in *D. takahashii* and also shows higher repeat density. Species-specific *de novo* repeat libraries were used for masking (see Methods).
