# Supplementary material for: Long-read genome assemblies for the study of chromosome expansion: Drosophila kikkawai, Drosophila takahashii, Drosophila bipectinata, and Drosophila ananassae
Source: G3 (Bethesda). 2023 Aug 23;13(10):jkad191. doi: 10.1093/g3journal/jkad191 (PMC10542312; doi:10.1093/g3journal/jkad191)
Supplement: jkad191_Supplementary_Data [file jkad191_supplementary_data.zip › Table_S1_G3-2023-404296.docx]

Table S1. Version information and references for the Bioinformatics tools used in this study

| **Tool** | **Version** | **URL** | **Reference** |
| --- | --- | --- | --- |
| 3D-DNA | 180419 | <https://github.com/aidenlab/3d-dna> | PMID: 28336562 |
| BBMap | 38.86 | <https://sourceforge.net/projects/bbmap/> |  |
| BlobTools2 | 3.2.7 | <https://blobtoolkit.genomehubs.org/> | PMID: 32071071 |
| BUSCO | 4.1.4 | <https://gitlab.com/ezlab/busco> | PMID: 34320186 |
| Canu | 2.1 | <https://github.com/marbl/canu> | PMID: 28298431 |
| Earl Grey | Git commit 2dcbd8b | <https://github.com/TobyBaril/EarlGrey> | DOI: 10.21203/rs.3.rs-1812599/v1 |
| Flye | 2.8.1 | <https://github.com/fenderglass/Flye> | PMID: 30936562 |
| GCpp | 1.0.0-1807624 | <https://github.com/PacificBiosciences/gcpp> |  |
| GEP UCSC Genome Browser | 435 | <https://gander.wustl.edu> |  |
| Hapo-G | Git commit ca2c732 | <https://github.com/institut-de-genomique/HAPO-G> | PMID: 33987534 |
| HiCExplorer | 3.6 | <https://github.com/deeptools/HiCExplorer> | PMID: 29335486 |
| LAST | 1406 | <https://gitlab.com/mcfrith/last> | PMID: 21209072 |
| NCBI BLAST+ | 2.13.0 | <https://blast.ncbi.nlm.nih.gov/Blast.cgi> | PMID: 20003500 |
| NextPolish | 1.3.0 | <https://github.com/Nextomics/NextPolish> | PMID: 31778144 |
| pbmm2 | 1.1.0 | <https://github.com/PacificBiosciences/pbmm2> |  |
| POLCA | 3.4.2 | <https://github.com/alekseyzimin/masurca> | PMID: 32589667 |
| Porechop | 0.2.4 | <https://github.com/rrwick/Porechop> |  |
| Purge Haplotigs | 1.1.1 | <https://bitbucket.org/mroachawri/purge_haplotigs/> | PMID: 30497373 |
| quickmerge | 0.3 | <https://github.com/mahulchak/quickmerge> | PMID: 27458204 |
| RepeatMasker | 4.1.2-p1 | <http://repeatmasker.org/> |  |
| SEQUELstats | Git commit 452194f | <https://github.com/VertebrateResequencing/SEQUELstats> |  |
| SequelTools | Git commit 553b9fe | <https://github.com/ISUgenomics/SequelTools> | PMID: 33004007 |
| SMRT Link | 7.0.1.66975 | <https://www.pacb.com/support/software-downloads/> |  |
| SPALN | 2.3.3f | <https://github.com/ogotoh/spaln> | PMID: 22848105 |
| UCSC Genome Browser | 435 | <https://hgdownload.soe.ucsc.edu/admin/exe/> | PMID: 36420891 |
| WU-BLAST | 2.0 | <https://blast.advbiocomp.com/> |  |
