# Supplementary material for: Long-read genome assemblies for the study of chromosome expansion: Drosophila kikkawai, Drosophila takahashii, Drosophila bipectinata, and Drosophila ananassae
Source: G3 (Bethesda). 2023 Aug 23;13(10):jkad191. doi: 10.1093/g3journal/jkad191 (PMC10542312; doi:10.1093/g3journal/jkad191)
Supplement: jkad191_Supplementary_Data [file jkad191_supplementary_data.zip › Table_S2_G3-2023-404296.docx]

**Table S2**. Number of putative Muller Element F scaffolds and the estimated size of the F Element in each *Drosophila* species with RefSeq assemblies.*

| Species | UCSC Database | # F Element Scaffolds | # *D. melanogaster* F Element Genes Placed in F Element Scaffolds | Estimated size of F Element |
| --- | --- | --- | --- | --- |
| *Drosophila melanogaster* | DmelRefSeq1 | 1 | 80 | 1348131 |
| *Drosophila mauritiana* | DmauRefSeq1 | 1 | 79 | 1160600 |
| *Drosophila sechellia* | DsecRefSeq1 | 1 | 79 | 1277805 |
| *Drosophila simulans* | DsimRefSeq3 | 1 | 79 | 1146867 |
| *Drosophila yakuba* | DyakRefSeq3 | 1 | 79 | 1429802 |
| *Drosophila santomea* | DsanRefSeq2 | 1 | 80 | 1429326 |
| *Drosophila teissieri* | DteiRefSeq1 | 1 | 80 | 1428144 |
| *Drosophila erecta* | DereRefSeq1 | 2 | 80 | 1465390 |
| *Drosophila ficusphila* | DficRefSeq2 | 1 | 77 | 1477415 |
| *Drosophila suzukii* | DsuzRefSeq2 | 1 | 78 | 2568912 |
| *Drosophila subpulchrella* | DspuRefSeq1 | 1 | 78 | 2259897 |
| *Drosophila biarmipes* | DbiaRefSeq2 | 1 | 78 | 1925543 |
| *Drosophila takahashii* | DtakRefSeq2 | 1 | 77 | 3447280 |
| *Drosophila eugracilis* | DeugRefSeq2 | 3 | 78 | 1180308 |
| *Drosophila rhopaloa* | DrhoRefSeq2 | 1 | 77 | 4365352 |
| *Drosophila elegans* | DeleRefSeq2 | 1 | 77 | 1954318 |
| *Drosophila kikkawai* | DkikRefSeq2 | 1 | 76 | 2258760 |
| *Drosophila serrata* | DserRefSeq1 | 3 | 77 | 2184026 |
| *Drosophila bipectinata* | DbipRefSeq2 | 2 | 75 | 20416075 |
| *Drosophila ananassae* | DanaRefSeq2 | 5 | 75 | 20019240 |
| *Drosophila pseudoobscura* | DpseRefSeq1 | 1 | 77 | 1881070 |
| *Drosophila persimilis* | DperRefSeq1 | 1 | 77 | 1944625 |
| *Drosophila miranda* | DmirRefSeq1 | 1 | 77 | 2366016 |
| *Drosophila guanche* | DguaRefSeq1 | 1 | 77 | 1279380 |
| *Drosophila subobscura* | DsobRefSeq1 | 1 | 77 | 1505893 |
| *Drosophila obscura* | DobsRefSeq2 | 1 | 77 | 1598006 |
| *Drosophila arizonae* | DariRefSeq1 | 4 | 72 | 2251440 |
| *Drosophila mojavensis* | DmojRefSeq2 | 1 | 72 | 1756888 |
| *Drosophila hydei* | DhydRefSeq1 | 3 | 71 | 1335917 |
| *Drosophila virilis* | DvirRefSeq1 | 4 | 70 | 1670261 |
| *Drosophila novamexicana* | DnovRefSeq1 | 3 | 74 | 2058893 |
| *Drosophila albomicans* | DalbRefSeq1 | 1 | 75 | 3955117 |
| *Drosophila grimshawi* | DgriRefSeq2 | 1 | 73 | 1266291 |
| *Drosophila busckii* | DbusRefSeq1 | 1 | 72 | 998951 |

* Two *Drosophila* species with RefSeq genome assemblies were omitted from the analysis to identify Muller Element F scaffolds. For *D. willistoni*, the Muller Element F has merged with Muller Element E. For *Drosophila navojoa*, the RefSeq assembly for Muller Element F is incomplete — genes located on the *Drosophila melanogaster* F Element can be placed in at least 50 scaffolds in the *D. navojoa* RefSeq assembly.
